# Supplementary material for: Mechanism and function of DNA replication‐independent DNA‐protein crosslink repair via the SUMO‐RNF4 pathway
Source: EMBO J. 2021 Aug 4;40(18):e107413. doi: 10.15252/embj.2020107413 (PMC8441304; doi:10.15252/embj.2020107413)
Supplement: Supplementary file 2 — Expanded View Figures PDF [file EMBJ-40-e107413-s002.pdf]

Expanded View Figures

Figure EV1. RNF4 promotes SUMO-dependent resolution of DNMT1 DPCs (related to Fig 1).

A Representative images from the experiment shown in Fig 1B. Scale bar, 10 μm.
B U2OS cells released from double thymidine synchronization in early S phase were pre-treated or not with SUMOi for 15 min, pulse-labelled with 5-azadC for 30 min and incubated in the presence or absence of SUMOi and ubiquitin E1 inhibitor (UBi). Cells collected at the indicated times were pre-extracted and immunostained with DNMT1 antibody. DNMT1 foci formation was analysed by quantitative image-based cytometry (QIBC; red bars, mean; > 4,000 cells analysed per condition). Data are representative of three independent experiments.
C Immunoblot analysis of HeLa cells transfected with non-targeting control (CTRL) or RNF4 siRNA. *, cross-reactive band.
D U2OS cells were transfected with non-targeting control (CTRL) or RNF111 siRNA. *RNF111* mRNA levels were analysed by qPCR. Primers to *GAPDH* were used as a normalization control (mean ± SEM; $n = 2$ independent experiments).
E HeLa cells transfected with indicated siRNAs were released from double thymidine synchronization in early S phase and treated with 5-azadC for 30 min, washed and collected at the indicated times. Soluble and chromatin-enriched fractions were immunoblotted with indicated antibodies.
F U2OS cells transfected with indicated siRNAs were released from double thymidine block, pulse-treated 2 h later with 5-azadC for 30 min and processed for QIBC analysis of DNMT1 foci counts as in (B) (red bars, mean; > 8,500 cells analysed per condition). Data are representative of three independent experiments.
G HAP1 cells (WT or independent derivative ΔRNF4 clones (#1–8 and #1–15) expressing a truncated form of RNF4 lacking the C-terminal RING domain) were synchronized in S phase by overnight treatment with thymidine. Following removal of thymidine, cells were treated with 5-azadC for 30 min and processed as in (E).
H Immunoblot analysis of indicated HAP1 cell lines showing truncation and reduced expression of endogenous RNF4.
I Immunoblot analysis of soluble and chromatin-enriched fractions of HeLa cells treated with formaldehyde and/or SUMOi as indicated.
J U2OS cells transfected with indicated siRNAs were treated with formaldehyde for 1 h. Cells were pre-extracted and fixed at the indicated times after formaldehyde removal, immunostained with SUMO2/3 antibody and processed for QIBC analysis of SUMO2/3 foci counts (red bars, median; dashed lines, quartiles; > 6,000 cells analysed per condition). Data show SUMO2/3 foci counts per cell normalized to the mean foci count of untreated cells and are representative of three independent experiments.
K As in (J), but showing total number of SUMO2/3 foci per cell.

© 2021 The Authors

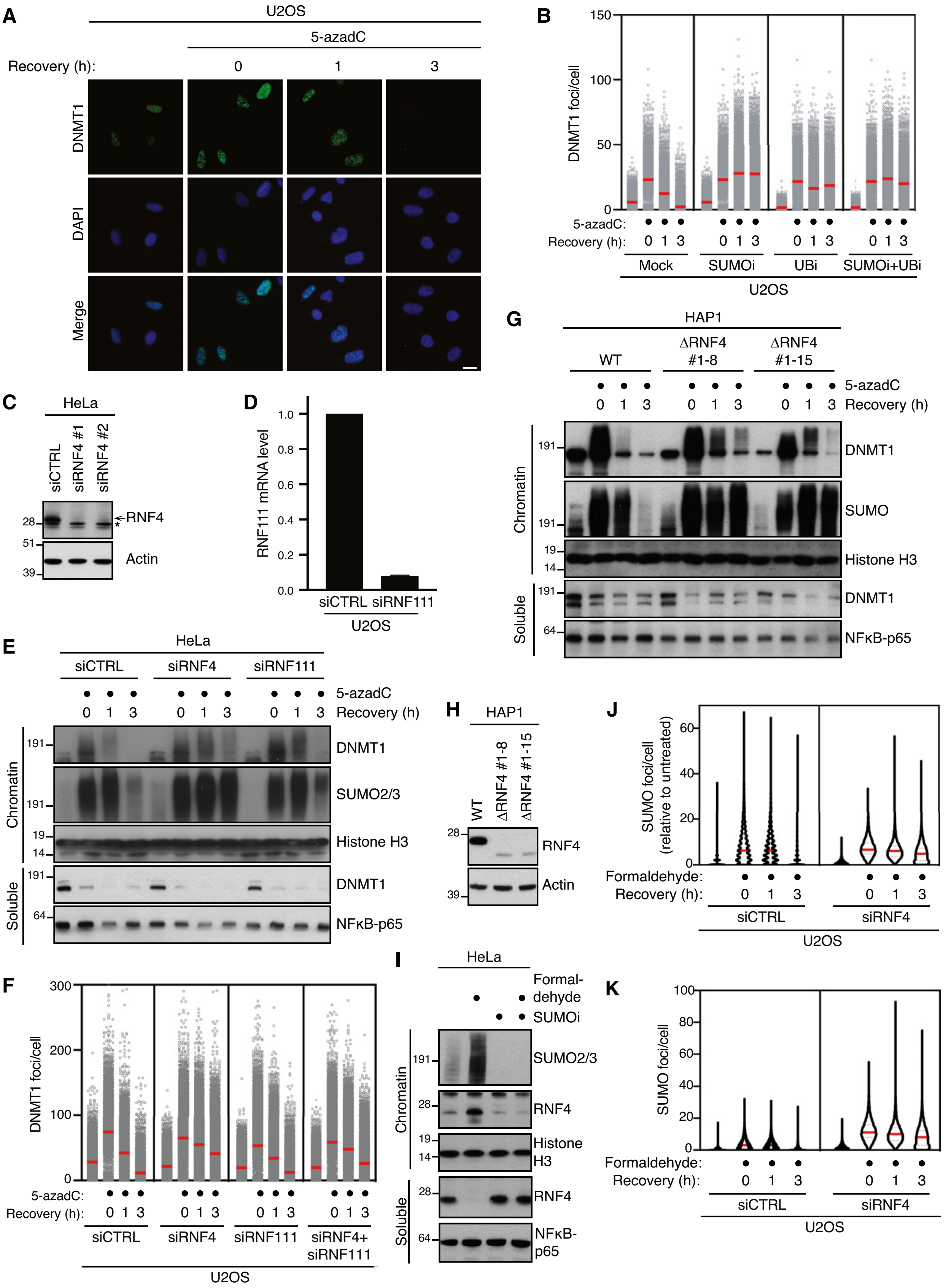
A
U2OS
5-azadC
Recovery (h):
0
1
3
DNMT1
DAPI
Merge
B
DNMT1 foci/cell
150
100
50
0
5-azadC:
Recovery (h):
0 1 3
Mock
SUMOi
UBi
SUMOi+UBi
U2OS
C
HeLa
siCTRL
siRNF4 #1
siRNF4 #2
28
51
39
RNF4
*
Actin
D
RNF111 mRNA level
1.0
0.8
0.6
0.4
0.2
0.0
siCTRL
siRNF111
U2OS
E
HeLa
siCTRL
siRNF4
siRNF111
5-azadC
Recovery (h)
Chromatin
Soluble
191
19
14
64
DNMT1
SUMO2/3
Histone H3
NFκB-p65
F
DNMT1 foci/cell
300
200
100
0
siRNF4+ siRNF111
G
HAP1
WT
ΔRNF4 #1-8
ΔRNF4 #1-15
SUMO
H
ΔRNF4 #1-8
ΔRNF4 #1-15
I
Formal-dehyde
SUMOi
Histone H3
NFκB-p65
J
SUMO foci/cell (relative to untreated)
60
40
20
Formaldehyde:
siRNF4
K
SUMO foci/cell
80
60

Figure EV1.

Figure EV2. PIAS4-dependent DPC SUMOylation (related to Fig 2).

A Sperm chromatin was left untreated or treated with 2,000 J/m^2 of UV-C and added to non-replicating egg extracts in the presence or absence of 10 μM of Talazoparib (PARPi). At the indicated time points, chromatin was recovered via chromatin spin-down and samples were immunoblotted with the indicated antibodies. Note that UV-C triggers PARylation at 2 min (lane 7), which is abolished in the presence of PARPi.

B Sperm chromatin was treated with 2,000 J/m^2 of UV-C and added to non-replicating egg extracts in the presence of PARPi. SUMO E1 inhibitor was added where indicated. Chromatin was recovered via chromatin spin-down and samples were immunoblotted with the indicated antibodies.

C NPE was either mock- or PIAS4-depleted, added to p4xDPC and analysed as in Fig 2A.

D Schematic representation of PIAS4 functional domains and mutations introduced into the SIM motifs (Kaur *et al*, 2017).

E NPE was either mock- or PIAS4-depleted and supplemented with WT PIAS4 or a mutant containing inactivating substitutions in the SIM1 and SIM2 domains (SIM1/2*) (D). Protein samples were immunoblotted with the indicated antibodies.

F Samples from (E) were added to p4xDPC^{SUMO} and analysed as in Fig 2A.

G Sperm chromatin was left untreated or exposed to 2,000 J/m^2 of UV-C and added to non-replicating egg extracts that were either mock- or PIAS4-depleted and supplemented with recombinant PIAS4 WT or SIM1/2*. PARPi was added where indicated. Chromatin was recovered via chromatin spin-down, and samples were immunoblotted with the indicated antibodies.

H Samples from Fig 2F were added to untreated sperm chromatin. At the indicated time points, chromatin was recovered via chromatin spin-down and samples were immunoblotted with the indicated antibodies.

I Samples from Fig 2F were added to UV-C-treated sperm chromatin in the presence of PARPi. At the indicated time points, chromatin was recovered via chromatin spin-down and samples were immunoblotted with the indicated antibodies.

J Immunoblot analysis of whole cell lysate of HeLa cells transfected or not with Myc-PIAS4 expression construct. Lysate from Myc-PIAS4-expressing cells was distributed equally between the three individual IP conditions in Fig 2J (800 μg per sample).

K HeLa/GFP-DNMT1 cells transfected with non-targeting control (CTRL) or PIAS4 siRNAs were treated with 5-azadC for the indicated times, collected and subjected to GFP immunoprecipitation under denaturing conditions, and immunoblotted with indicated antibodies.

L Immunoblot analysis of HeLa/GFP-DNMT1 cells transfected with non-targeting control (CTRL) or PIAS4 siRNAs.

M U2OS cells were transfected with control (CTRL) or PIAS4 siRNA targeting the 3′UTR and subsequently transfected with plasmids encoding WT or catalytically inactive (CI) Myc-PIAS4 expression plasmid. Cells were then exposed to 5-azadC, fixed 1 h later and co-immunostained with Myc and DNMT1 antibodies. Representative images are shown. Note that Myc-PIAS4 CI is recruited to DNMT1 DPCs even in the absence of endogenous PIAS4 activity. Scale bar, 5 μm.

N Immunoblot analysis of U2OS cells transfected with control (CTRL) or PIAS4 siRNA targeting the 3′UTR.

 © 2021 The Authors

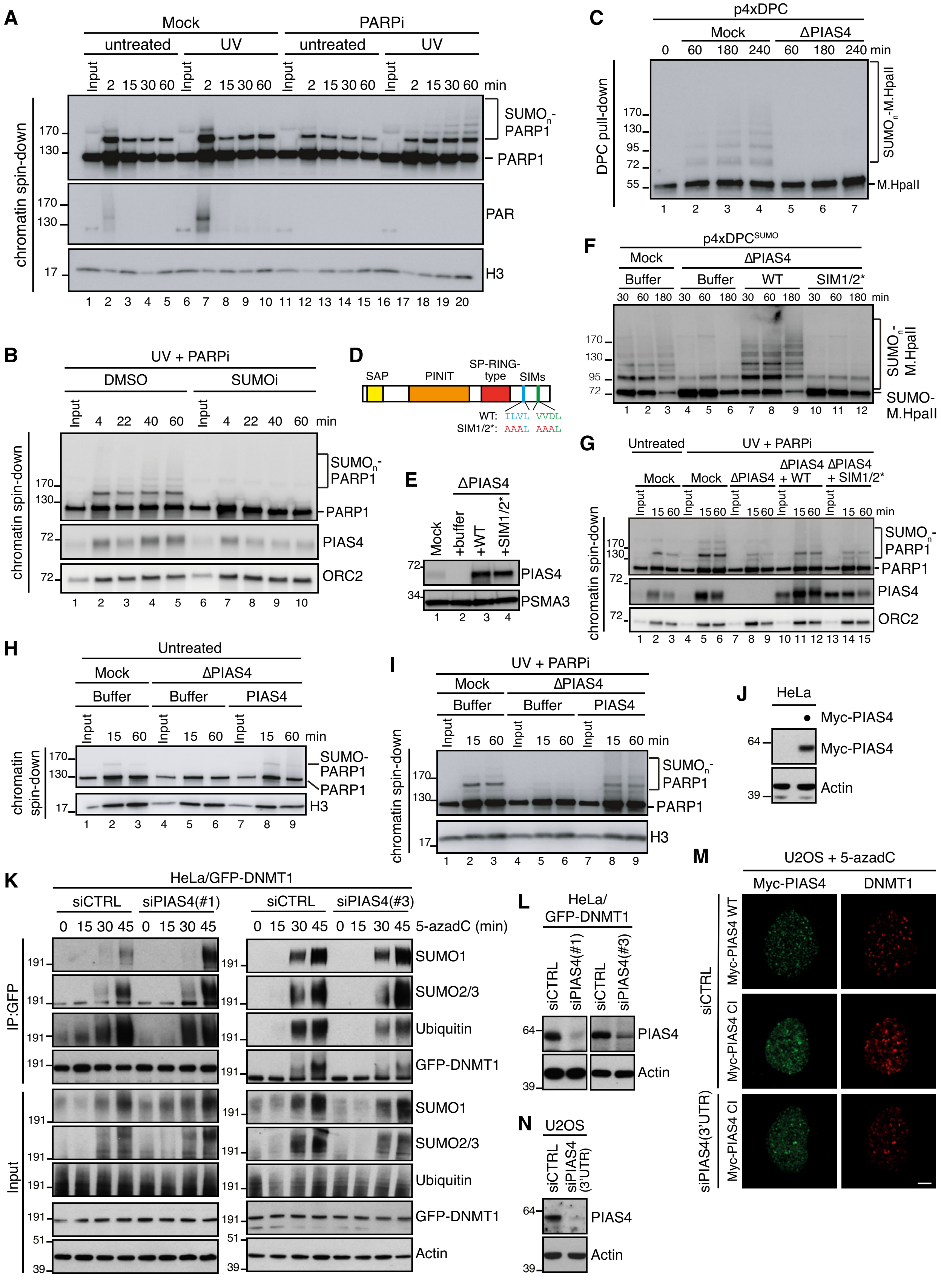
A
Mock
PARPi
untreated
UV
untreated
UV
Input 2 15 30 60 Input 2 15 30 60 Input 2 15 30 60 Input 2 15 30 60 min
chromatin spin-down
SUMOn-PARP1
PARP1
PAR
H3
1 2 3 4 5 6 7 8 9 10 11 12 13 14 15 16 17 18 19 20
B
UV + PARPi
DMSO
SUMOi
Input 4 22 40 60 Input 4 22 40 60 min
SUMOn-PARP1
PARP1
PIAS4
ORC2
C
p4xDPC
Mock
ΔPIAS4
0 60 180 240 60 180 240 min
DPC pull-down
SUMOn-M.HpaII
M.HpaII
D
SAP
PINIT
SP-RING-type
SIMs
WT: ILVL VVDL
SIM1/2*: AAAL AAAL
E
ΔPIAS4
Mock
+buffer
+WT
+SIM1/2*
PIAS4
PSMA3
F
p4xDPCSUMO
Mock
ΔPIAS4
Buffer
Buffer
WT
SIM1/2*
30 60 180 30 60 180 30 60 180 30 60 180 min
SUMOn-M.HpaII
SUMO-M.HpaII
G
Untreated
UV + PARPi
Mock
Mock
ΔPIAS4
ΔPIAS4 + WT
ΔPIAS4 + SIM1/2*
Input 15 60
SUMOn-PARP1
PARP1
PIAS4
ORC2
H
Untreated
Mock
ΔPIAS4
Buffer
Buffer
PIAS4
Input 15 60 Input 15 60 Input 15 60 min
chromatin spin-down
SUMO-PARP1
PARP1
H3
I
UV + PARPi
Mock
ΔPIAS4
Buffer
Buffer
PIAS4
Input 15 60 Input 15 60 Input 15 60 min
SUMOn-PARP1
PARP1
H3
J
HeLa
Myc-PIAS4
Actin
K
HeLa/GFP-DNMT1
siCTRL
siPIAS4(#1)
siCTRL
siPIAS4(#3)
0 15 30 45 0 15 30 45 0 15 30 45 0 15 30 45 5-azadC (min)
IP:GFP
Input
SUMO1
SUMO2/3
Ubiquitin
GFP-DNMT1
Actin
L
HeLa/GFP-DNMT1
siCTRL
siPIAS4(#1)
siCTRL
siPIAS4(#3)
PIAS4
Actin
M
U2OS + 5-azadC
Myc-PIAS4
DNMT1
siCTRL
Myc-PIAS4 WT
Myc-PIAS4 CI
siPIAS4(3'UTR)
Myc-PIAS4 CI
N
U2OS
siCTRL
siPIAS4 (3'UTR)
PIAS4
Actin

Figure EV2.

Figure EV3. Impact and modification of SUMOylated DPCs in *Xenopus* egg extracts (related to Fig 3).

A p2xDPC^{Leads} was replicated in egg extracts in the presence of radiolabelled nucleotides in the presence or absence of the SUMO E1 or ubiquitin E1 inhibitors. Samples were analysed by native agarose gel electrophoresis as in Fig 3B. Red arrowheads indicate open circular molecules (OC) that have not yet undergone repair.

B Samples from (A) were recovered by DPC pull-down and immunoblotted against M.HpaII. Note that in the presence of the ubiquitin E1 inhibitor DPC ubiquitylation and degradation by both SPRTN and the proteasome are severely inhibited (Duxin *et al*, 2014; Larsen *et al*, 2019). Residual ubiquitylation likely reflects ubiquitin E1 enzymes in the extract activated prior to addition of the inhibitor.

C Scheme illustrating DPC repair in egg extracts when M.HpaII or SUMO-M.HpaII is crosslinked to ssDNA (pDPC^{ssDNA} or pDPC^{SUMOssDNA}). In this setting, DPC ubiquitylation is mainly driven by the E3 ubiquitin ligase RFWD3 and does not require DNA replication (Gallina *et al*, 2021).

D pDPC^{ssDNA} or pDPC$^{SUMO\text{-}ssDNA}$ were incubated in non-replicating egg extracts. DPCs were recovered by pull-down and immunoblotted against M.HpaII. Note the similar kinetics of degradation for SUMOylated and non-SUMOylated DPCs.

E Indicated volumes of whole egg CSF-arrested extract and NPE were immunoblotted with RNF4 antibody next to a dilution series of recombinant His-RNF4. A band migrating around 28 kDa and immunodepleted with the RNF4 antibody (Fig 3H) is indicated as RNF4. * denotes non-specific bands that are not immunodepleted by the RNF4 antibody.

F Samples from Fig 3E were immunoblotted with the indicated antibodies.

G p4xDPC^{SUMO} was incubated in NPE or CSF-arrested extracts. At the indicated times, the DPCs were recovered and immunoblotted against M.HpaII.

H Indicated volumes of whole egg CSF-arrested extract and NPE were immunoblotted with PIAS4 and PSMA3 antibodies. Where indicated, CSF-arrested extract was supplemented with 10 ng/μl of recombinant PIAS4.

I p4xDPC^{SUMO} was incubated with CSF-arrested extracts from (H). DPCs were recovered at the indicated time points and immunoblotted against M.HpaII.

J p4xDPC^{SUMO} was incubated in NPE for 60 min and supplemented with buffer or either 7 ng/μl or 35 ng/μl (5×) of recombinant RNF4 (WT) in the presence of MG262 where indicated. Protein samples were immunoblotted with the indicated antibodies.

K Samples from (J) were recovered via DPC pull-down at the indicated time following RNF4 addition and immunoblotted against M.HpaII.

L, M Independent replicates of Fig 3L.

N Sperm chromatin was incubated for 1 h in CSF-arrested egg extract in the presence or absence of 10 μM of the pan-CDK inhibitor R547. Note that chromosome condensation is inhibited in the presence of R547, consistent with CDK inhibition. Scale bar, 30 μm.

O Sequential addition of *Xenopus* egg extracts to DPC-containing plasmids. After the first addition of NPE, the SUMOylated DPCs were recovered and incubated in CSF-arrested egg extract in the presence or absence of R547. The DPCs were again recovered and immunoblotted against M.HpaII.

© 2021 The Authors

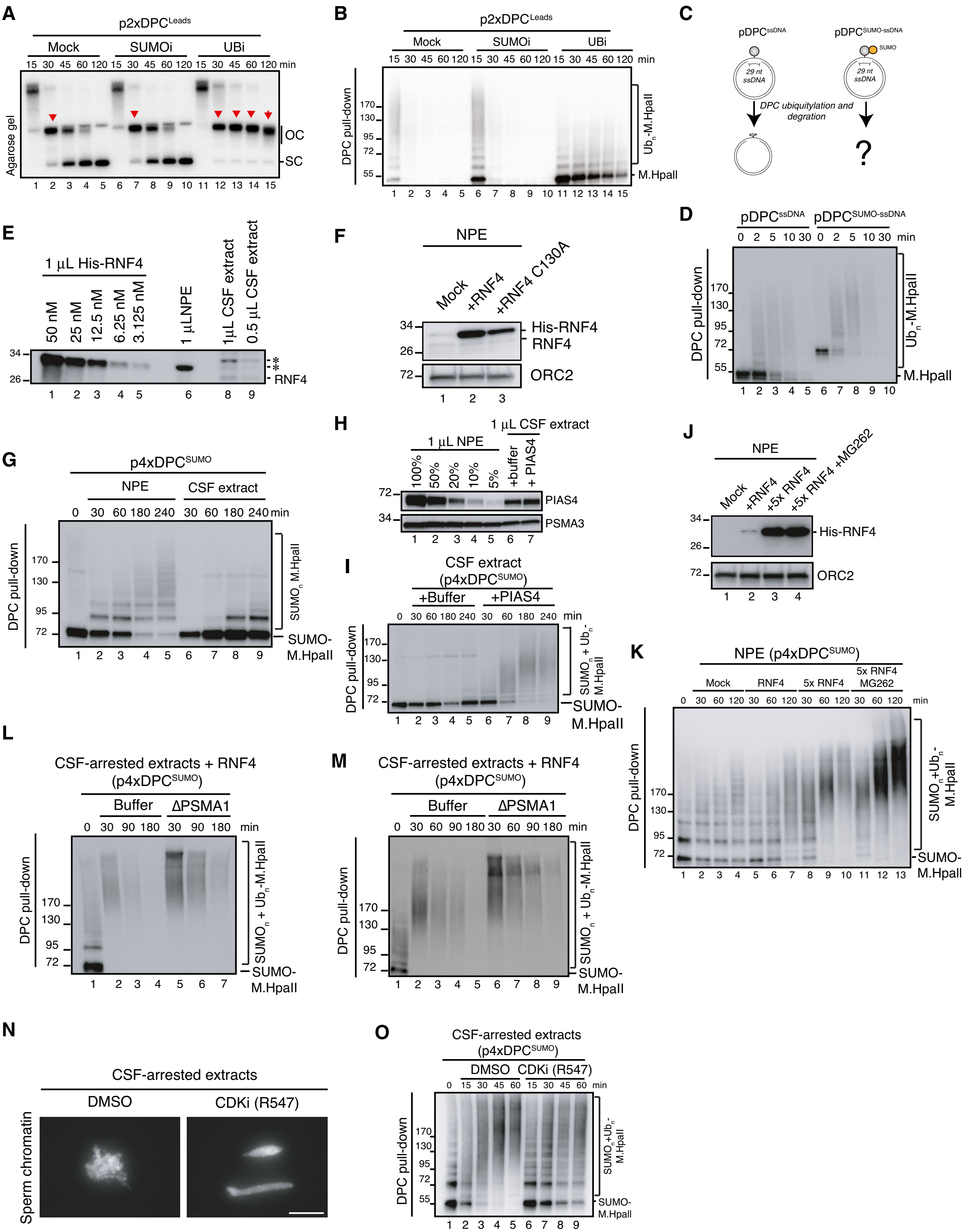
A
p2xDPC^Leads
Mock SUMOi UBi
15 30 45 60 120 15 30 45 60 120 15 30 45 60 120 min
Agarose gel
OC
SC
B
p2xDPC^Leads
Mock SUMOi UBi
DPC pull-down
Ubn-M.HpaII
M.HpaII
C
pDPC^ssDNA
pDPC^SUMO-ssDNA
SUMO
29 nt ssDNA
DPC ubiquitylation and degration
?
D
pDPC^ssDNA pDPC^SUMO-ssDNA
0 2 5 10 30 0 2 5 10 30 min
DPC pull-down
Ubn-M.HpaII
M.HpaII
E
1 µL His-RNF4
50 nM 25 nM 12.5 nM 6.25 nM 3.125 nM
1 µLNPE
1µL CSF extract
0.5 µL CSF extract
RNF4
F
NPE
Mock +RNF4 +RNF4 C130A
His-RNF4
RNF4
ORC2
G
p4xDPC^SUMO
NPE CSF extract
0 30 60 180 240 30 60 180 240 min
DPC pull-down
SUMOn-M.HpaII
SUMO-M.HpaII
H
1 µL NPE
1 µL CSF extract
100% 50% 20% 10% 5% +buffer + PIAS4
PIAS4
PSMA3
I
CSF extract (p4xDPC^SUMO)
+Buffer +PIAS4
0 30 60 180 240 30 60 180 240 min
DPC pull-down
SUMOn + Ubn-M.HpaII
SUMO-M.HpaII
J
NPE
Mock +RNF4 +5x RNF4 +5x RNF4 +MG262
His-RNF4
ORC2
K
NPE (p4xDPC^SUMO)
Mock RNF4 5x RNF4 5x RNF4 MG262
0 30 60 120 30 60 120 30 60 120 30 60 120 min
DPC pull-down
SUMOn+Ubn-M.HpaII
SUMO-M.HpaII
L
CSF-arrested extracts + RNF4 (p4xDPC^SUMO)
Buffer ΔPSMA1
0 30 90 180 30 90 180 min
DPC pull-down
SUMOn + Ubn-M.HpaII
SUMO-M.HpaII
M
CSF-arrested extracts + RNF4 (p4xDPC^SUMO)
Buffer ΔPSMA1
0 30 60 90 180 30 60 90 180 min
DPC pull-down
SUMOn + Ubn-M.HpaII
SUMO-M.HpaII
N
CSF-arrested extracts
DMSO CDKi (R547)
Sperm chromatin
O
CSF-arrested extracts (p4xDPC^SUMO)
DMSO CDKi (R547)
0 15 30 45 60 15 30 45 60 min
DPC pull-down
SUMOn+Ubn-M.HpaII
SUMO-M.HpaII

Figure EV3.

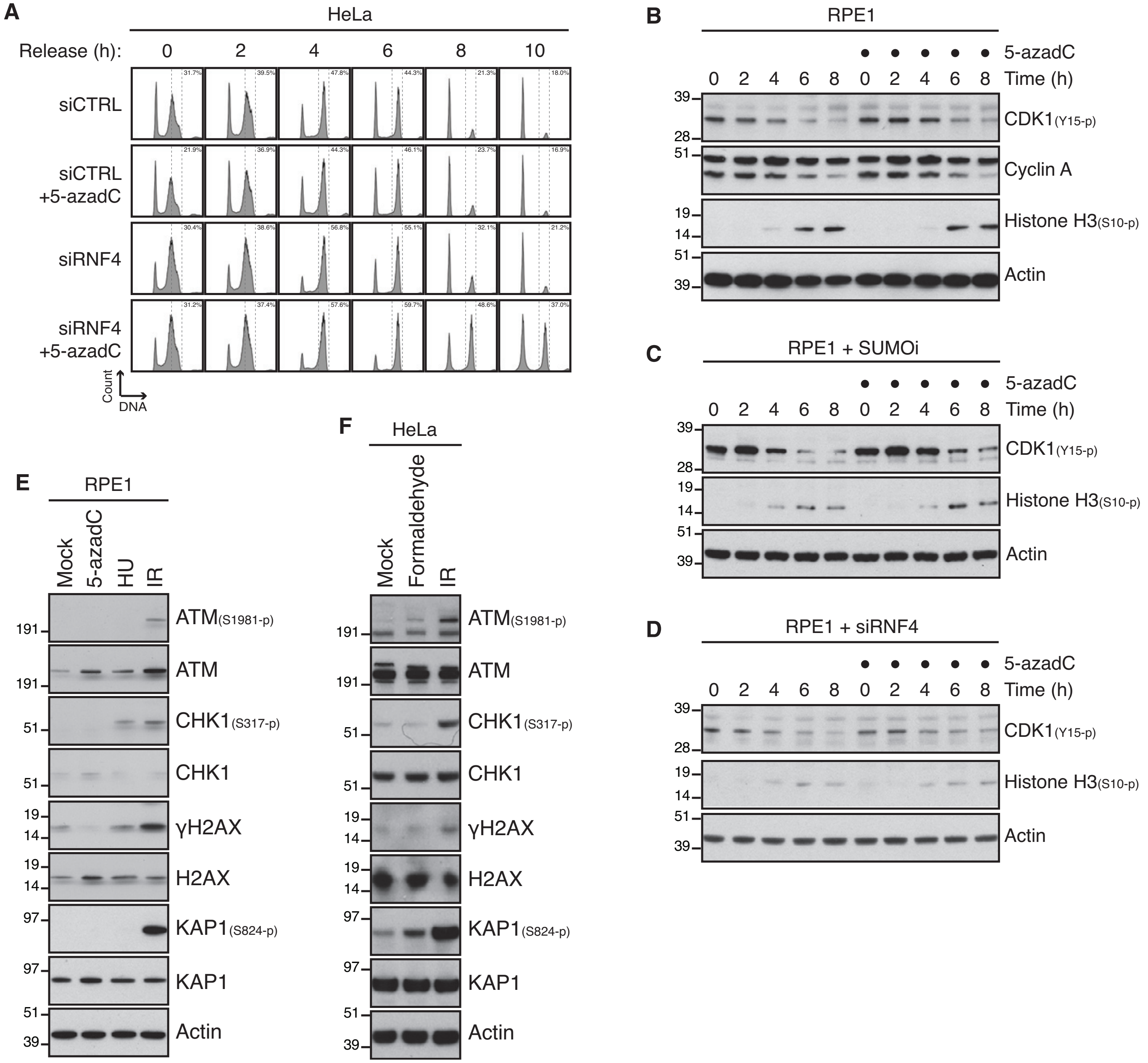

Figure EV4. DPCs in uninterrupted duplex DNA do not activate a DNA damage checkpoint (related to Fig 4).

A HeLa cells were transfected with indicated siRNAs and synchronized in early S phase by double thymidine block. Following release from the block, cells were pulse-labelled with 5-azadC in late S phase for 30 min. Cells were then collected at the indicated times after 5-azadC withdrawal and analysed by flow cytometry. Data are representative of three independent experiments. Proportion of cells with G2/M DNA content is indicated.

B RPE1 cells were synchronized in early S phase by double thymidine block. Following release from the block, cells were pulse-labelled or not with 5-azadC for 30 min in late S phase, incubated with nocodazole, collected at the indicated times and immunoblotted with indicated antibodies.

C As in (B), except that SUMOi was added to the culture medium 15 min prior to 5-azadC treatment.

D As in (B), except that cells were transfected with RNF4 siRNA prior to double thymidine block synchronization.

E Immunoblot analysis of RPE1 cells exposed to indicated genotoxic agents and collected 1 h later.

F Immunoblot analysis of HeLa cells exposed to indicated genotoxic agents and collected 1 h later.

 © 2021 The Authors

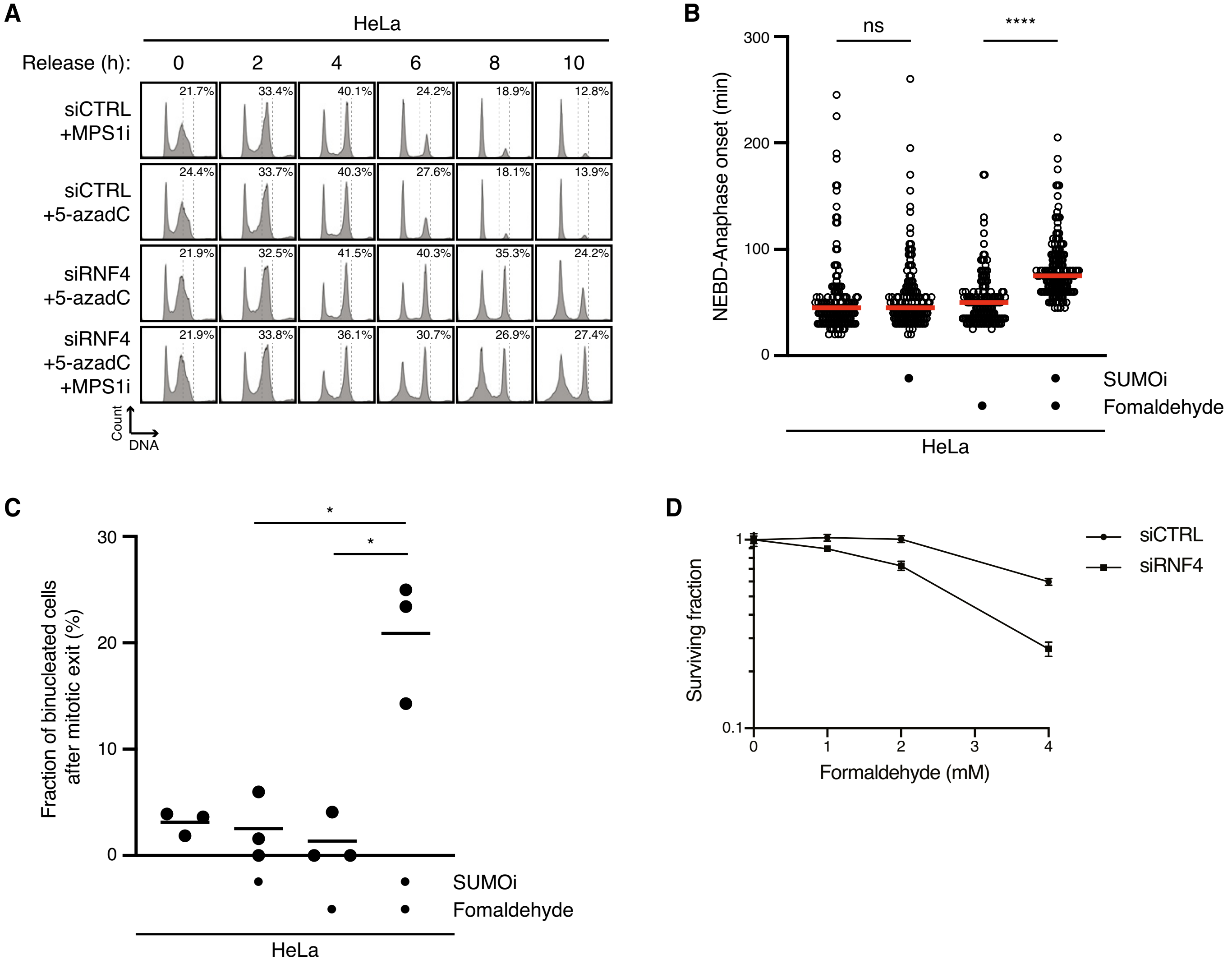

Figure EV5. Unresolved DPCs undermine faithful mitotic chromosome segregation and cellular fitness (related to Fig 5).

A HeLa cells transfected with indicated siRNAs were treated or not with 5-azadC for 30 min and/or MPS1i in late S phase. Cells were then collected at the indicated times after 5-azadC withdrawal and analysed by flow cytometry. Data are representative of three independent experiments. Proportion of cells with G2/M DNA content is indicated.

B HeLa cells were synchronized in early S phase by double thymidine block, released and pulse-treated 7 h later with formaldehyde for 1 h in the presence or absence of SUMOi. Following formaldehyde removal, cells were subjected to live-cell imaging analysis, and the duration of mitosis (nuclear envelope breakdown (NEBD) to anaphase onset) was quantified (red bars, median; at least 156 cells, pooled from three independent experiments, were analysed per condition; ****$P < 0.0001$, ns: not significant, Mann–Whitney test).

C Quantification of mitotic defects in cells in (B) (black bars, mean; $n = 3$ independent experiments; > 150 cells quantified per condition; *$P < 0.05$, paired t-test).

D Clonogenic survival of HeLa cells transfected with indicated siRNAs and subjected to indicated doses of formaldehyde for 30 min before replating (mean ± SEM; $n = 2$ independent experiments).
